# The use of stem cells and organoids for modeling host-microbe interactions in low-biomass tissues

**DOI:** 10.3389/fcimb.2025.1641366

**Published:** 2025-08-20

**Authors:** Claire A. Shaw, Margo Verstrate, Kinga Graniczkowska, Katie R. Risoen, Pouya Dini, Bart C. Weimer

**Affiliations:** ^1^ School of Veterinary Medicine, Population Health and Reproduction, University of California, Davis, Davis, CA, United States; ^2^ 100K Pathogen Genome Project, University of California, Davis, Davis, CA, United States

**Keywords:** low-biomass microbiota, stem cells, host/microbe interactions, microbiome models, *in vitro* model

## Abstract

Stem cells and organoids have emerged as pivotal biological tools for biologically relevant models. Together, these *in vitro* models realistically recapitulate structural and functional elements of the *in vivo* organ, allowing for studies of cellular, molecular, and genetic features that underpin various diseases that are difficult to observe in low-biomass tissues. Stem cells, and more recently organoids, have been applied *in vivo* as regenerative therapies. The emergence of the microbiome as an occupant throughout different body locales requires new approaches to understand the complex cellular interactions with the host tissue at each site. The success of regenerative medicine strategies and therapeutic development is intricately linked to this understanding and management of host–microbe dynamics. Interactions with the host microbiome and infections can both significantly impair tissue regeneration and compromise the function of stem cell–derived therapies. Therefore, a comprehensive understanding of how pathogens and the microbiome interact with stem cells and organoids is relevant for developing safe and effective regenerative medicine interventions. This review explores the evolving landscape of organoid technology, including a discussion on the importance of stem cell studies and considerations for organoid development that are important for use as models to study microbiome interactions. Additionally, this work describes the pivotal role of cell culture models in advancing host–microbe interaction studies in understudied low-biomass organs such as the stomach and reproductive tract. Through this assessment, we aim to shed light on the potential of these models to transform the approach to studying and managing infectious diseases within the context of regenerative medicine.

## Introduction

Stem cells are foundational to the success of regenerative medicine and relevant to a myriad of diseases. Their ability to differentiate into specialized cell types makes them invaluable for treating a wide range of diseases, including neurodegenerative disorders, cardiovascular diseases, and autoimmune conditions (Kolagar et al., 2020; Lerman and Lerman, 2021). Organoids, three-dimensional *in vitro* cultures that mimic the structure and function of native organs, represent a significant advancement in stem cell research for their ability to mimic cells in organs (Kol et al., 2014; Kretzschmar and Clevers, 2016; Zhao et al., 2022). These organoids, which can be derived from either adult stem cells (ASCs) or pluripotent stem cells, can be grown to resemble various organs with multicellular organization (Pain, 2021; Han et al., 2024; Pasca et al., 2024). This advancement provides a foundational platform for studying organ development, disease modeling, and drug testing, and for bridging the gap between traditional cell cultures and *in vivo* models (Jensen and Teng, 2020; Weng et al., 2023; Verstegen et al., 2025). They faithfully recapitulate structural and functional elements of the *in vivo* organ, allowing for studies of atypical cellular, molecular, and genetic features that underscore diseases (Lancaster and Huch, 2019; Andrews and Kriegstein, 2022; Chen et al., 2023; Silva-Pedrosa et al., 2023). Furthermore, organoids hold promise for cell replacement approaches to injury or disease (Wu et al., 2023) in humans and animals (Nakamura and Sato, 2018; Penning and van den Boom, 2023). While tissue repair is a well-known use of regenerative medicine approaches, the emergence of a microbiome in many internal tissues brings about a new challenge in understanding microbial interactions in various organs, especially in tissues that were once thought to be ‘sterile.’ The success of regenerative medicine strategies is intricately linked to understanding and managing host–pathogen interactions (Kol et al., 2014; Mohamad-Fauzi et al., 2023). *In vitro* models to study the association of the microbiome that adequately reflect the complexity of *in vivo* responses are lacking, thereby creating a gap in understanding how microbes impact various tissues that were once thought to be sterile (Yi et al., 2022; Hugon and Golos, 2023; Suarez Arbelaez et al., 2023). Infections can significantly impair tissue regeneration and compromise the function of stem cell-derived therapies (Mikulska et al., 2014), yet it is unclear what the role of the microbiome is related to therapeutic protocols. Therefore, a comprehensive understanding of how microbes interact with stem cells and organoids is crucial for developing safe and effective regenerative medicine interventions.

This review explores the evolving landscape of organoid technology, including a discussion on the importance of stem cell functions and current approaches to organoid development. This work also includes considerations of the pivotal role for cell culture models in advancing host–microbe interaction studies in low-biomass organ systems. By examining recent advancements, challenges, and future directions, we aim to provide insights into how stem cell and organoid models are reshaping our understanding of host–pathogen dynamics, disease progression, and therapeutic advancements.

## Stem cell models and bacterial infections

Stem cells serve as the foundational cell type for organoid development, providing the regenerative capacity and cellular diversity required to model complex tissues and organ systems *in vitro* (Clevers et al., 2014). Their ability to self-renew and differentiate makes them indispensable for recreating the architecture and functionality of human organs. However, this intrinsic plasticity and susceptibility to environmental signals also make stem cells key targets for microbial interactions, including bacterial infections and microbiome associations to recapitulate *in vivo* assays (Kolb-Maurer et al., 2004; Hess and Rambukkana, 2015; Wizenty and Sigal, 2023). Unexpectedly, the association of common gut bacteria induces changes in the immune status (Hou et al., 2017) and differentiation trajectory (Mohamad-Fauzi et al., 2023) of stem cells without inducing apoptosis, suggesting that the microbiome may change the activity of stem cells *in vivo* and further highlights that microbes have direct access to stem cells *in vivo*. Understanding these host–pathogen interactions is critical, as pathogens can exploit stem cells to establish infections, alter their differentiation pathways, and potentially disrupt organoid integrity (Mohamad-Fauzi et al., 2023).

Carriage of bacteria in stem cells is of particular concern in regenerative medicine, which employs stem cell therapies as a treatment for a multitude of diseases, including digestive disorders, autoimmune liver disease, arthritis, and some cancers (Hoang et al., 2022). An emerging consideration is how microbiome members condition the developing immune system in early life via interactions with stem cells (Mishra et al., 2021). Insights into these interactions not only enhance the fidelity of organoid models for microbiome research but also provide a deeper understanding of how stem cells contribute to innate immunity and tissue resilience in both physiological and pathological conditions.

A diverse set of pathogenic, commensal, and opportunistic bacteria can interact with and influence stem cell function. The mechanisms of interaction include direct contact, such as bacterial adherence and invasion, and more indirect effects mediated by secreted toxins, metabolites, and signaling molecules. The consequences of these interactions are multidimensional; they can impair or enhance the regenerative capacity of stem cells, alter differentiation trajectories, and in some cases, contribute to disease progression, including chronic inflammation and tumorigenesis (Marrazzo et al., 2019; O’Rourke and Kempf, 2019; Pani, 2025). Understanding the spectrum of microbial influence on stem cells is essential for optimizing their clinical application and for developing strategies to mitigate microbial interference in stem cell–based therapies.

Mesenchymal stem cells (MSCs), somatic multipotent stromal cells, play an emerging role in regenerative medicine due to their ability to migrate to sites of injury, differentiate into multiple lineages, and secrete a wide range of immunomodulatory factors (Wang et al., 2014; O’Rourke and Kempf, 2019). However, these same properties render MSCs highly responsive to environmental cues, including microbial signals at mucosal interfaces such as the gut lumen (Marrazzo et al., 2019; Mohamad-Fauzi et al., 2023). These interactions with pathogenic and commensal microbes influence the regenerative and immunological behavior of MSCs, ultimately affecting the clinical outcomes of MSC-based therapies.

Microbial exposure has been shown to significantly influence MSC behavior. Multiple gastrointestinal bacteria have been shown to adhere and invade MSCs without impacting cell survival, including pathogenic *Salmonella enterica* serovar Typhimurium and probiotic *Lactobacillus acidophilus*, indicating the potential for bacterial carriage in stem cells (Kol et al., 2014). Though bacterial association did not affect stem cell survival, co-incubation of canine MSCs with these gastrointestinal bacteria altered MSC immunoregulatory profiles through induction of cytokine transcription, modification of surface markers such as CD54, and enhancement of prostaglandin E2 (PGE_2_)–mediated suppression of T-cell proliferation (Kol et al., 2014). Additionally, *S.* Typhimurium association inhibited MSC migration, which is notable given migration is a key feature for their therapeutic application (Kol et al., 2014). Such findings demonstrate that bacterial interactions can manipulate MSC function through subtle regulatory shifts rather than overt cytotoxicity and apoptosis, with implications for both therapeutic efficacy and microbial persistence in host tissues.

The ability of microbes to influence stem cell activity is further emphasized in the context of chronic infections. Hematopoietic stem cells (HSCs) are among the most common cell types used in stem cell–based therapies yet are particularly influenced by chronic infection (Li et al., 2023). Chronic inflammation and chronic infection have both been shown to deplete HSC populations, in part through an increase in the terminal differentiation pathway (Matatall et al., 2016). *Mycobacterium tuberculosis* has also been shown to reprogram HSCs in the bone marrow via activation of the type I interferon (IFN-I) axis (Khan et al., 2020). This reprogramming disrupts iron acquisition and metabolism, impairs myelopoiesis, and ultimately hinders the immune response to *M. tuberculosis* infections (Khan et al., 2020). Notably, this hindered HSC activity persisted a year after initial infection, suggesting that microbial exposure can exert prolonged immunological consequences that extend beyond acute disease (Khan et al., 2020).

Microbial interactions have also been shown to disrupt differentiation pathways. Exposure of human and goat MSCs to *S.* Typhimurium interfered with trilineage differentiation, particularly inhibiting osteogenic and chondrogenic differentiation (Mohamad-Fauzi et al., 2023). Moreover, *S.* Typhimurium activated anti-apoptotic and pro-proliferative signaling in host cells, a function that favors pathogen survival while compromising host tissue regeneration (Mohamad-Fauzi et al., 2023).

In contrast with their susceptibility to microbial influence, MSCs also exhibit intrinsic antimicrobial activity, suggesting the potential for dual therapeutic roles. For mice infected with virulent *Klebsiella pneumonia*, the administration of placental MSCs (PMSCs) led to a significant reduction in bacterial load via localized recruitment of polymorphonuclear neutrophils (PMNs) and concurrent dampening of potentially harmful T-cell and natural killer (NK) cell responses (Wang et al., 2020). Additionally, MSC-derived antimicrobial peptides inhibited methicillin-resistant *S. aureus* (MRSA) biofilm development in chronic *Staphylococcus aureus* infections. When combined with antibiotics, this approach evoked synergetic activity toward bacterial clearance and improved wound healing (Chow et al., 2020). These findings underscore the complex, bidirectional nature of MSC–microbe interactions and position MSCs as both potential targets of microbial manipulation and active participants in host defense.

While stem cells hold significant clinical promise, their vulnerability to bacterial influence underscores a key gap in our understanding of microbiome–stem cell interactions across different organ systems. The complexity of these interactions necessitates model systems that can isolate and dissect these processes in controlled conditions, as traditional *in vivo* models are often too complex to resolve these interactions at a mechanistic level. *In vitro* work aimed at disentangling these dynamics, such as that using organoid models, can bridge the experimental gap and support translational findings.

## Considerations for the development of organoid models

Often described as “mini-organs in a dish,” organoids are defined by their three-dimensional (3D) multicellular structure and ability to self-organize, self-renew, and recapitulate key structural, functional, and molecular features of native tissues and organs (Clevers, 2016). They offer significant advantages over traditional 2D cell cultures, including long-term culture viability, enhanced cellular complexity, and preserved cell–cell and cell–matrix interactions (Kim et al., 2020). Organoids have been successfully generated for a wide range of mammalian species and multiple organs, including the skin, brain, liver, stomach, gastrointestinal tract, heart, pancreas, testis, endometrium, and placenta (Turco et al., 2018; Brooks et al., 2021; Puschhof et al., 2021b; Cho et al., 2022; Gnecco et al., 2023; Sreenivasamurthy et al., 2023; Tian et al., 2023; Stopel et al., 2024). The development of an organoid model includes deciding on multiple key components, including stem cell origin, scaffold, and media. These elements collectively influence the variability, heterogeneity, and functionality of the organoids, ultimately determining their suitability for specific applications.

**Figure 1:**
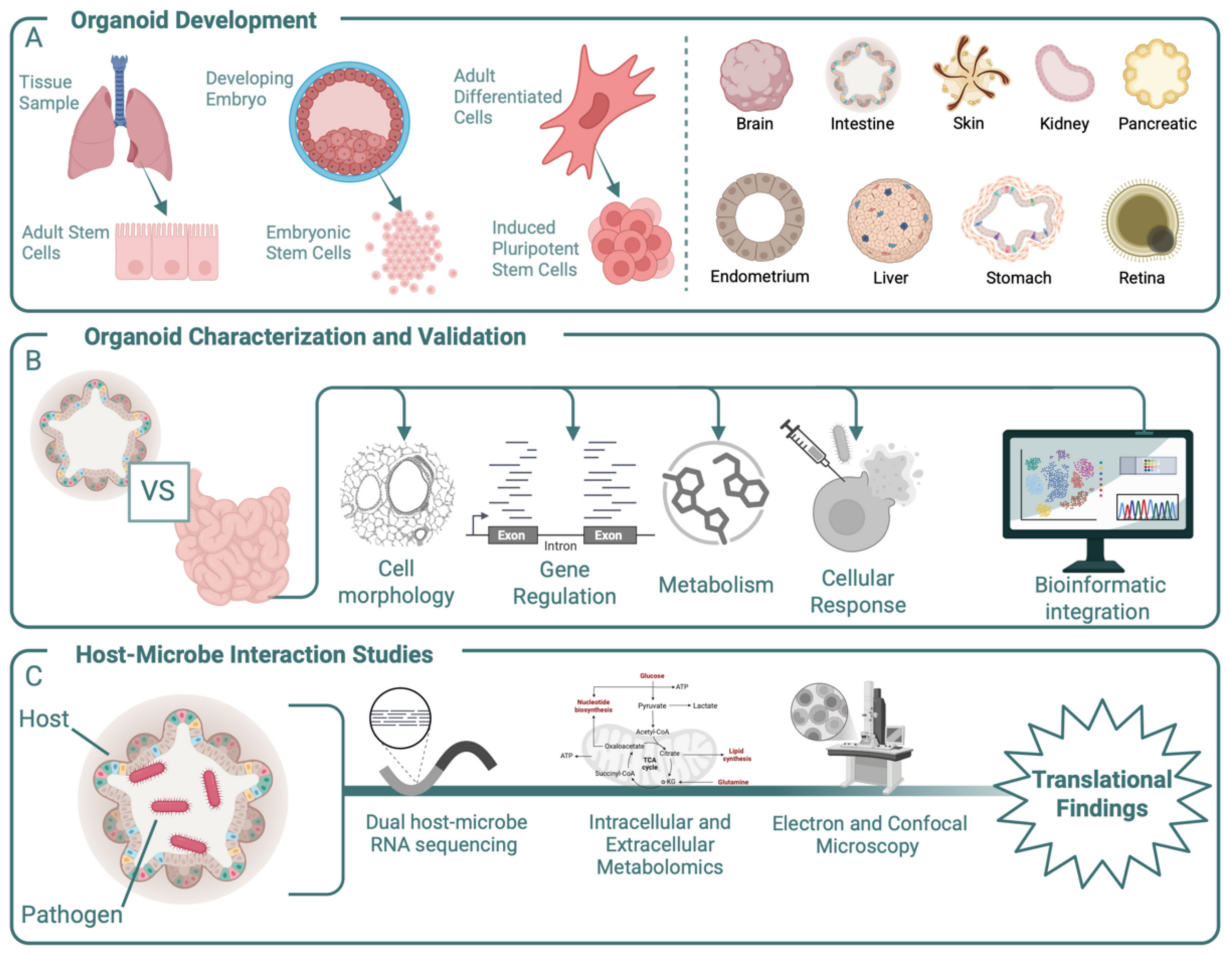
Overview of the development, characterization, and experimental use of organoids. **(A)** Organoids can be derived from multiple cell sources, including primary tissue samples, embryonic cells, and differentiated adult cells. *In-vitro* models for multiple organs have been developed and used as *in-vitro* models. **(B)** Once developed, organoids should be compared to relevant *in-vivo* tissue using multiple approaches that include morphological, genetic, and metabolic testing to ensure *in-vitro* responses recapitulate *in-vivo* ones. The ability of an organoid to replicate an *in-vivo* response to exogenous material such as drugs or microbes should also be considered prior to novel experimentation. These tests require complex bioinformatic integration to fully capture the organoid’s capabilities. **(C)** After characterization, organoids can be used to uncover mechanistic responses to experimental conditions such as the addition of a bacterial pathogen. Outputs for these experiments include genetic regulation, metabolic activity, and cell–cell interactions. Together these experimental findings can aid *in-vivo* outcomes.

Organoids can be derived from three cell sources: ASCs, embryonic stem cells (ESCs), or induced pluripotent stem cells (iPSCs) (**Figure 1**) (Dutta et al., 2017; Ouchi et al., 2019; Pleguezuelos-Manzano et al., 2020) (**Figure 1A**). ASC-derived organoids, which are predominately epithelial and lack vasculature, closely resemble mature tissues and retain the genetic and functional characteristics of the donor. This makes them particularly suitable for disease modelling, personalized medicine approaches, and microbiome association studies with mature tissue structures (Yang et al., 2023). However, ASC-derived organoids lack the ability to produce diverse cell types in one model and are challenging to culture long term or at large scales (Yang et al., 2023). ESCs, derived from the inner cell mass of blastocysts, and iPSCs, generated by reprogramming of somatic cells, offer the advantage of producing organoids with greater cellular diversity, including mesenchymal, epithelial, and endothelial cells. Compared to ASC-derived organoids, which are typically unified in cell type, iPSCs can simultaneously differentiate into multiple cell types—such as spinal cord neurons and skeletal muscle cells—creating complex hybrid structures representative of *in vivo* tissue arrangements (Yang et al., 2023). While both the diversity and differentiation capacity enhance their value for tissue developmental studies, ESC- and iPSC-derived organoids often do not reach full maturity, failing to fully replicate the functionality of adult tissues with mature and differentiated cell types. However, organoids that contain a mixture of differentiated and non-differentiated stem cells may be a faithful model for *in vivo* inflammation, where epithelial degradation leads to the exposure of stem cells, as seen in gut inflammation and intestinal stem cells (Quan et al., 2025).

Beyond the selection of cell type, there are also multiple scaffolding materials and media components to choose from. For some cell sources, like iPSCs, cells are embedded in an extracellular matrix (ECM) that mimics the tissue-specific microenvironment. An ECM provides structural support and biochemical cues for organoid growth and maintenance, enabling the spherical structure inherent to organoids. Matrigel, derived from mouse Engelbreth-Holm-Swarm sarcoma, is a widely used basement membrane ECM, mainly composed of laminin, collagen IV, entactin, perlecan, and growth factors (Hughes et al., 2010). Despite its effectiveness, Matrigel has notable limitations, including its animal origin, batch-to-batch variability, and poorly defined composition, and the ability of bacteria to digest Matrigel complicates its use in host–microbe interaction studies (Hughes et al., 2010; Kozlowski et al., 2021; Kim et al., 2022). Alternatives to Matrigel are emerging, including natural hydrogels, synthetic hydrogels, and hybrid hydrogels (Kozlowski et al., 2021; Kim et al., 2022). Additionally, non-hydrogel systems such as silk microspheres and suspension cultures, where organoids are cultured in microwells designed and coated for low adhesion, have emerged (Matkovic Leko et al., 2023). Last, the cell culture media delivers essential signals that guide organoid differentiation, growth, and maintenance. Organoid culture media consist of basal media, antibiotics/antimycotics, and soluble factors that mimic the native tissue microenvironment (**Figure 1**) (Pleguezuelos-Manzano et al., 2020). This combination of cell source, scaffold, and culture media together dictates the success of an organoid model and contributes to its replicability, making them crucial considerations during the development stage.

While organoids have transformed our ability to model tissue biology *in vitro*, their initial simplicity left gaps in physiological relevance. A critical leap in this field is the development of assembloids: advanced models that incorporate multiple cell types (e.g., stromal, vascular, and immune components), mimicking the cellular cross talk seen in native tissues (Kanton and Pasca, 2022). This integration enables studies of more complex tissue environments, from tumor-stroma interactions to the influence of bacterial interactions, providing insights that were previously inaccessible. The use of assembloids in host–microbe interactions allows precise experimentation; however, their geometry and orientation must be considered prior to use. In traditional organoid cultures, the cells’ apical surface faces inward (apical-in), forming a closed lumen (Puschhof et al., 2021b). While ideal for some studies, this orientation obstructs direct access to the epithelial surface, a challenge for research on host–pathogen interactions, where the apical surface is the primary site of contact. To address this, polarity reversal techniques have been developed, flipping the apical surface outward (apical-out) (Co et al., 2019). This innovation eliminates the need for micromanipulation techniques, allowing organoids to be directly exposed to infectious agents or therapeutic compounds in the biologically relevant orientation.

Organoids are a promising platform, but their utility is not without limitations. Reproducibility remains a significant challenge, with variability stemming from both inter- and intra-organoid heterogeneity, as well as temporal changes during development and across passages. Current protocols lack standardization, with batch-to-batch variation often exacerbated by the reliance on inconsistent animal-derived matrices (Kozlowski et al., 2021; Kim et al., 2022). Another shortcoming lies in the absence of vascularization and immune components in many current organoid models. Without blood vessels, nutrient and oxygen delivery becomes diffusion-limited, leading to within-organoid heterogeneity, especially in larger organoids (Grebenyuk et al., 2023; Tan et al., 2024). Similarly, the lack of immune cells within the organoid diminishes the ability to study immune responses or inflammation-driven diseases (Jeong and Kang, 2023).

Thorough validation of the organoids through characterization of the structural, molecular, and functional level is also an essential step to validate their relevance, reproducibility, and suitability for specific applications (**Figure 1B**). To better reflect *in vivo* conditions, organoid models also need to capture contributions of the local microbiome, but to date the incorporation of a microbiome in cell culture models is not standard practice and remains difficult to do (Allen and Sears, 2019; Zhong et al., 2023). Though organoids are not a research panacea and still have many shortcomings, they present a promising method for reducing the need for animal models and for increasing the repeatability of study findings. By leveraging the benefits of stem cell and organoid models, researchers can discern facets of pathogen behavior, track host cell responses, and better understand disease progression with unprecedented precision.

## Understanding host–microbe interactions in stem cell and organoid models

One area where early stem cell models and complex organoids have proven particularly valuable is the study of host–microbe interactions in low-biomass situations—often intricate and dynamic relationships with profound implications for health and disease. Commensal microbes are increasingly linked to improved immune function, conditioning the immune system with exposure to bone marrow, the promotion of systemic health, and resistance to infections. Contrastingly, pathogenic microbes remain a global health challenge, driving outbreaks and undermining regenerative cell therapies, underscoring the urgent need for effective treatment strategies. Understanding these dual dynamics is paramount, and organoids have emerged as a critical platform for this purpose. Because organoids provide a controlled and largely reproducible *in vitro* environment, these models enable precise examination of microbial interactions and host responses. Additionally, organoids allow for time series experiments, which provide valuable insights that are largely unattainable *in vivo*.

**Figure 2:**
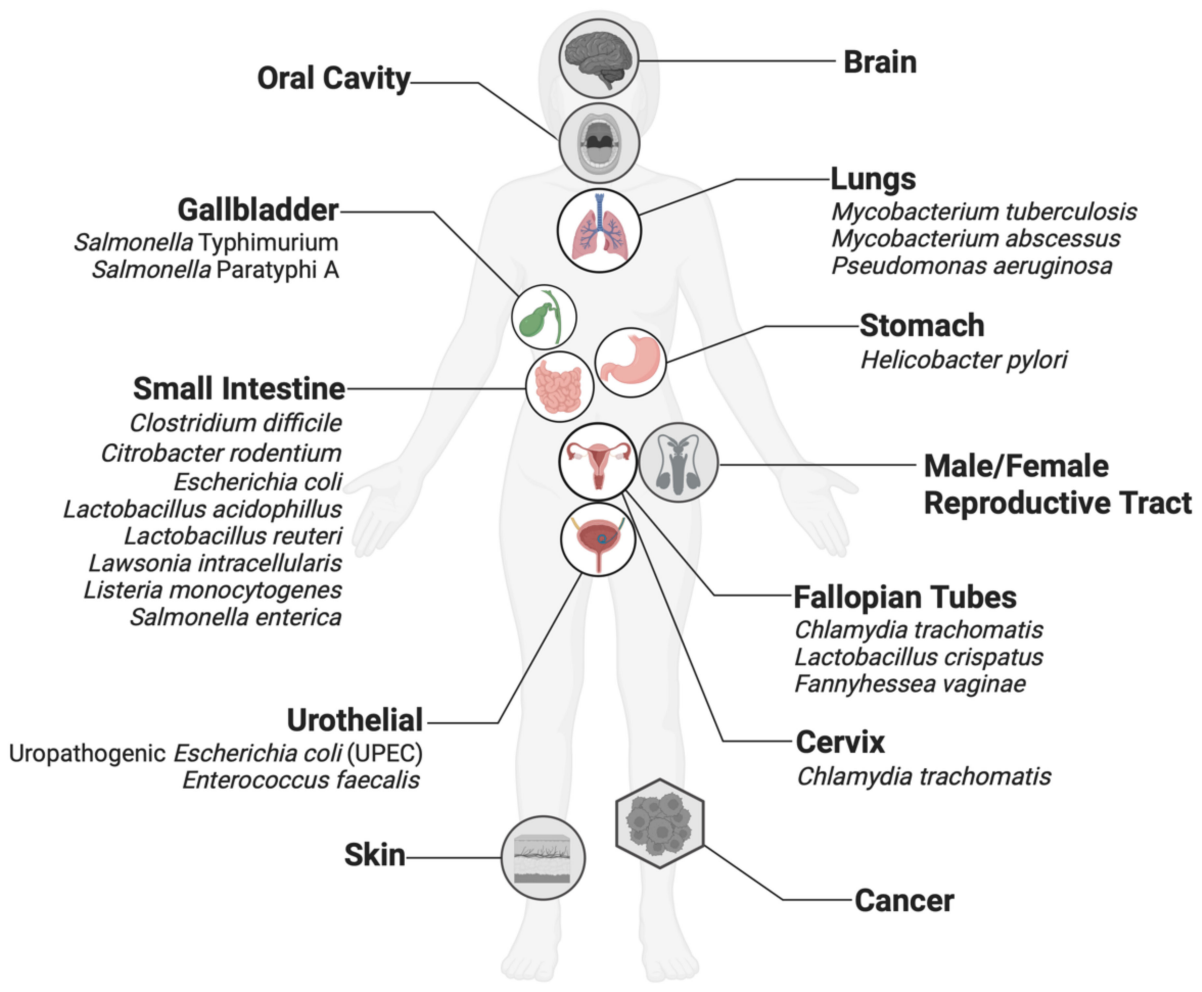
Organoid models have been developed for many organs thought to harbor low-biomass microbiomes, such as the stomach and reproductive tracts. Organs in white circles have been used in host-microbe interaction studies with the microbes listed here and are discussed in this review. Organs in gray circles have been developed but are not routinely used in host-microbe studies and are not discussed in this review.

Importantly, organoid-based studies enable the modelling of both high-biomass systems, characterized by abundant microbial populations, and typically understudied low-biomass systems, where microbial presence is sparse and thus challenging to model (**Figure 2**). While organoid models have proven particularly useful for modeling host–microbe interactions in microbially dense organs, the scope of this review is limited to body locales presumed to have lower microbial density and those that are understudied (Puschhof et al., 2021a; Xiang et al., 2024).

Many connections between microbes and diseases remain merely correlative, largely due to the challenges of unraveling the multitude of changes during host–microbe interactions in a reductionist yet meaningful manner. This highlights the need for advanced *in vitro* model systems to provide mechanistic insights into microbial impacts on the host tissue that have translational importance for therapeutic value. Despite their limitations, animal models and conventional 2D cell cultures are fundamental tools in deciphering host–pathogen mechanisms during bacterial infections. Organoid models present a valuable alternative to animal models, which demand significant resources such as funding, labor, and housing facilities. In addition to reducing costs, organoids offer new insights into disease development mechanisms and the impact of virulence factors on host epithelium by closely replicating *in vivo* tissue environments. Furthermore, organoids provide ample opportunity to collect material for various downstream analyses, including that for metagenomics, metabolomics, and visualization (**Figure 1**), that can be used to provide new insights into mechanistic causes of infection that may serve as new therapeutic targets.

Notable progress has been made in understanding interactions in certain host contexts, such as gut microbes and gastrointestinal organoids derived from human (Chakrabarti et al., 2021; Puschhof et al., 2021b) and animal tissues (Beaumont et al., 2021). Nevertheless, studies focusing on low-biomass tissues remain relatively underexplored. The section below highlights organoids representing low-biomass tissue and details the work of their use in understanding microbial interactions in these contexts (**Table 1**).

**Table 1:** Organoid models of host–microbe interactions in different species and organs.

Organ	Origin	Model	Microbe	Reference
Lung	human	microinjection	*Mycobacterium tuberculosis*	(Iakobachvili et al., 2022a)
	human	microinjection	*Mycobacterium abscessus*	(Iakobachvili et al., 2022a)
	human	microinjection	*Pseudomonas aeruginosa*	(Tang et al., 2022)
	human	microinjection	*Pseudomonas aeruginosa*	(Bagayoko et al., 2021)
Stomach	mouse & human	microinjection	*Helicobacter pylori*	(Bertaux-Skeirik et al., 2015)
	human	microinjection	*Helicobacter pylori*	(Bartfeld et al., 2015)
	human	microinjection	*Helicobacter pylori*	(McCracken et al., 2014)
	human	suspension	*Helicobacter pylori*	(Huang et al., 2015)
	human	microinjection & monolayer	*Helicobacter pylori*	(Aguilar et al., 2022)
	mouse	microinjection & fragmentation	*Helicobacter pylori*	(Nascakova et al., 2024)
Gallbladder	mouse	suspension	*Salmonella Typhimurium*	(Scanu et al., 2015)
	human	monolayer	*Salmonella* Paratyphi A	(Sepe et al., 2020)
Small intestine	porcine	monolayer	Enterotoxigenic *E. coli* (ETEC)	(Vermeire et al., 2021)
	porcine	monolayer	*Lawsonia intracellularis*	(Resende et al., 2020)
	bovine	monolayer	*Salmonella* Dublin	(Kawasaki et al., 2024)
	chicken	suspension	*Lactobacillus acidophilus*	(Pierzchalska et al., 2017)
	chicken	suspension	*Salmonella enterica*	(Nash et al., 2021)
	chicken	suspension	*Salmonella* Typhimurium	(Lacroix-Lamande et al., 2023)
	chicken	suspension	*Salmonella* Typhimurium	(Mitchell et al., 2024)
	human	microinjection	*Escherichia coli O157:H7 and commensal E. coli*	(Karve et al., 2017)
	human	microinjection	*Clostridium difficile*	(Leslie et al., 2015)
	human	suspension	*Salmonella* Typhimurium *& Listeria monocytogenes*	(Co et al., 2019)
	mouse & human	microinjection	*Salmonella* Typhimurium	(Forbester et al., 2018)
	mouse	suspension	*Salmonella* Typhimurium *& Lactobacillus acidophilus*	(Lu et al., 2020)
	mouse	suspension	*Lactobacillus reuteri & Citrobacter rodentium*	(Wu et al., 2020)
	mouse	microinjection	*Salmonella* Typhimurium	(Wilson et al., 2015)
Urothelial	human	unspecified	Uropathogenic *Escherichia coli* (UPEC)	(Smith et al., 2006)
	human	fragmentation	*Enterococcus faecalis*	(Horsley et al., 2018)
	human	microinjection	Uropathogenic *Escherichia coli* (UPEC)	(Sharma et al., 2021)
Fallopian tube	mouse	suspension	*Chlamydia trachomatis*	(Rajeeve et al., 2020)
	human	fragmentation	*Chlamydia trachomatis*	(Kessler et al., 2019)
	human	suspension	*Lactobacillus crispatus*	(Yu et al., 2024)
	human	suspension	*Fannyhessea vaginae*	(Yu et al., 2024)
Cervix	human	suspension	*Chlamydia trachomatis*	(Koster et al., 2022)

### Stomach

Gastric organoids, which self-organize into gland and pit domains, have emerged as a valuable model system for studying the gastric pathogen *Helicobacter pylori* (Bartfeld et al., 2015). *H. pylori* infect the gastric mucosa and affect approximately half of the world’s population. *H. pylori* possess various virulence factors, including cytotoxin-associated gene A (*cagA*) and vacuolating cytotoxin (*vacA*), which contribute to its pathogenicity and are thought to be associated with progression to cancer (de Brito et al., 2019). While many infected individuals remain asymptomatic, *H. pylori* is a significant risk factor for peptic ulcers, gastric adenocarcinoma, and mucosa-associated lymphoid tissue (MALT) lymphoma (de Brito et al., 2019).

Microinjection of *H. pylori* into spherical gastric organoids has enabled a greater understanding of the interaction of *H. pylori* with the human (Bartfeld et al., 2015) and mouse gastric mucosa (Bertaux-Skeirik et al., 2015; Nascakova et al., 2024). Consistent with findings *in vivo*, gastric organoids infected with *H. pylori* induced robust epithelial responses such as c-Met receptor phosphorylation and increased epithelial cell proliferation within 24h (McCracken et al., 2014). Further confirming the discriminant response of gastric organoids and highlighting the importance of specific genes in disease, *cagA-*deficient *H. pylori* failed to produce the same epithelial response as wild-type *H. pylori* (McCracken et al., 2014). CD44 also plays a crucial role in *H. pylori*-induced epithelial cell proliferation and gastric carcinogenesis. *H. pylori* infection, particularly through the aforementioned *cagA*, stimulates the formation of a CagA/CD44/c-Met complex, leading to increased epithelial proliferation (Bertaux-Skeirik et al., 2015).

Work with gastric organoids has also revealed that metabolites released by human gastric organoids rapidly attract *H. pylori* (Huang et al., 2015). *H. pylori’s* chemoreceptor TlpB is highly sensitive to urea, enabling simultaneous detection and metabolism of urea gradients originating from the human gastric epithelium, allowing bacterial movement into and detection within the host epithelium (Huang et al., 2015). Once at the host interface, *H. pylori* exhibit a preference for attaching to highly differentiated pit cells within the gastric epithelium (Aguilar et al., 2022). Further studies demonstrated that infection of antrum-derived gastric organoid cells with *H. pylori* led to an increase in the expression of the stem cell marker leucine-rich repeat-containing G-protein coupled receptor 5 (Lgr5), suggesting that *H. pylori* infection may influence stem cell dynamics, potentially contributing to epithelial repair processes or, conversely, to pathological conditions such as cancer initiation (Nascakova et al., 2024).

### Small intestine

Replicating the small intestinal epithelium *in vitro* remains challenging; however organoids offer a promising alternative to reduce the cellular complexity without losing multicellular organization and communication completely. Small intestinal organoids, also called enteroids, retain the cell type diversity, spatial organization, and functional properties of the native epithelium, including crypt-villus architecture, barrier formation, and innate immune activity.

Porcine enteroids have been used extensively to model enteric infections and have demonstrated fidelity to *in vivo* responses. Consistent with diarrheal pathophysiology *in vivo*, porcine enteroids responded to enterotoxigenic *Escherichia coli* (ETEC) exposure with fluid secretion, altered ion transport, and increased expression of pro-inflammatory cytokines such as IL-8 (Vermeire et al., 2021). Moreover, when cultured as 2D monolayers, these enteroids allowed direct visualization and quantification of bacterial adhesion, revealing strain-specific adherence patterns. Human intestinal organoids (iHIOs), derived from pluripotent stem cells, have similarly enabled detailed analysis of human-specific pathogens. iHIOs have been used to model *E. coli* O157:H7 infection, demonstrating significant epithelial damage, loss of barrier integrity, and robust activation of inflammatory signaling pathways (Karve et al.). Infection also induced actin cytoskeletal rearrangement and promoted neutrophil chemotaxis, mimicking clinical features of hemorrhagic colitis (Karve et al., 2017). In contrast, commensal *E. coli* strains failed to elicit these effects, underscoring the specificity of pathogenic mechanisms and validating the organoid model’s discriminatory capacity.

Organoid systems have also proven effective across species, broadening their utility in comparative and translational studies. Bovine enteroids, for example, have been used to investigate *Salmonella* Dublin, a zoonotic pathogen that causes systemic disease in cattle and humans (Kawasaki et al., 2024). Chicken enteroids have also been used to explore *Salmonella* dynamics, with one study illustrating that wild-type *Salmonella* strains rapidly invaded and replicated within chicken enteroids, while mutant strains lacking virulence factors failed to establish infection (Sutton et al., 2023). Antimicrobial strategies and feed additives have also been tested in chicken enteroid models. A combination of organic acids and essential oils (OA+EO) added to chicken enteroids attenuated inflammatory cytokine production and preserved epithelial integrity during *Salmonella* infection (Mitchell et al., 2024). Notably, chicken enteroids derived from longitudinal intestinal regions differed in their susceptibility to *Salmonella* Typhimurium, highlighting regional variation in epithelial permissiveness and confirming the ability of organoids to faithfully replicate *in vivo* tissue regions (Lacroix-Lamande et al., 2023).

Organoids have also enabled in-depth modeling of pertinent pathogens that target the small intestine, like *Clostridium difficile* and *Salmonella* species. Using microinjection techniques, one group demonstrated that toxin-producing *C. difficile* strains cause pronounced epithelial destruction in colonic organoids, mimicking the mucosal damage observed in patients with pseudomembranous colitis (Leslie et al., 2015). Murine-derived enteroids have also helped clarify the role of innate immune factors in epithelial defense. Paneth cell-derived α-defensins were shown to significantly reduce *Salmonella* Typhimurium translocation and preserve tight junction integrity (Wilson et al., 2015). Enteroid systems not only provide a valuable model for dissecting the mechanisms of toxin-mediated injury but also offer a platform for evaluating immune responses and for testing therapeutic agents that could protect or restore epithelial integrity.

Potential therapeutics like probiotics and dietary modulators are being tested using enteroid models. In murine enteroids, *Lactobacillus acidophilus* and *L. reuteri* has been shown to attenuate *Salmonella*-induced epithelial damage, modulate inflammatory responses, and promote crypt regeneration (Lu et al., 2020; Wu et al., 2020). These effects are mediated, at least in part, through modulation of the Wnt/β-catenin signaling pathway—a key regulator of intestinal stem cell renewal and epithelial repair. In avian enteroid models, *L. acidophilus* combined with TLR2 ligands significantly stimulated epithelial proliferation and growth (Pierzchalska et al., 2017). This synergistic effect was linked to increased expression of stem cell markers and improved organoid viability, indicating a direct influence of probiotic signals on stem cell niche function. As organoid systems continue to evolve, they are likely to play a central role in refining probiotic therapies and understanding host–microbe interactions at the cellular and molecular levels in the small intestine.

### Gallbladder

Chronic *Salmonella* infection has been increasingly associated with gallbladder carcinoma (GBC), though the mechanisms linking infection to tumorigenesis are not yet fully understood. Gallbladder organoids provide a powerful tool to dissect these interactions at the cellular and molecular levels. Gallbladder organoids infected with *Salmonella* exhibit early features of malignant transformation, including loss of epithelial cohesion and polarity, as well as nuclear atypia characterized by enlarged, irregular nuclei with prominent nucleoli (Scanu et al., 2015).

Further contributing to the complexity of bacterial-driven tumorigenesis are the potential contributions of geographical distribution and genetic background. Investigation of the molecular landscape of GBC across different populations revealed that tumors from Indian patients—where *Salmonella typhi* is endemic—frequently harbored *S. typhi* DNA, TP53 mutations, and c-MYC overexpression (Scanu et al., 2015). In contrast, such alterations were infrequent in GBC samples from the Netherlands, suggesting a geographically linked, microbe-associated oncogenic signature. To model this *in vitro*, the researchers utilized gallbladder organoids derived from *Apc*^+/min mice, as well as mouse embryonic fibroblasts (MEFs) engineered to mimic the Indian patient’s genetic profile via TP53 inactivation and c-MYC overexpression. Remarkably, *Salmonella* infection of these organoids recapitulated key aspects of the Indian GBC molecular phenotype, including transformation-associated morphological changes and proliferative signaling. These findings underscore the utility of organoids in modeling infection-driven carcinogenesis and illustrate how they may be useful platforms for modeling the effect of genetic backgrounds on disease progression (Scanu et al., 2015).

**Figure 3:**
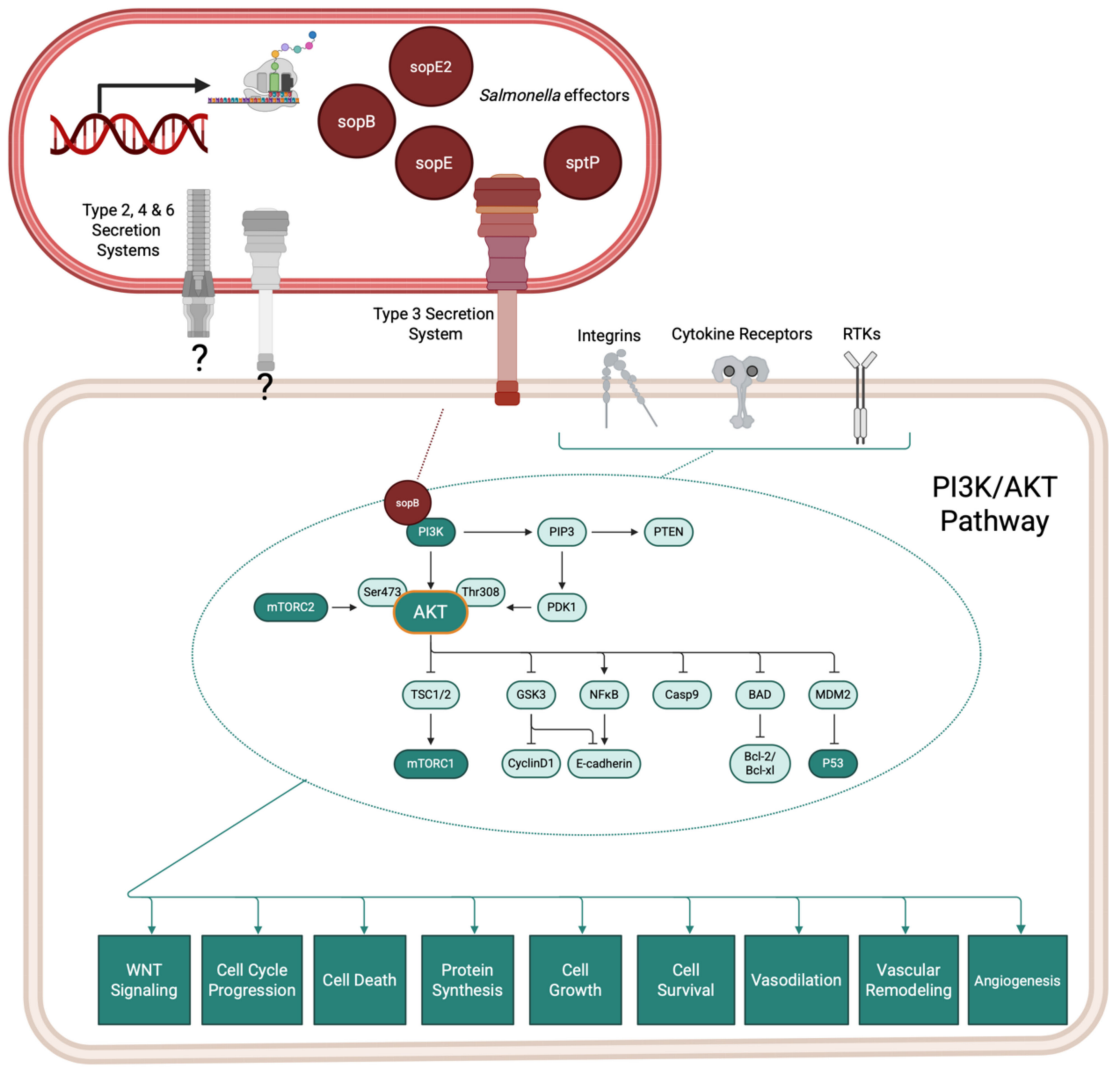
Salmonella infection of the gallbladder induces host PI3K/AKT signaling cascades which contribute to dysregulated cell cycling and cancer progression. Salmonella species encode effector proteins that contribute to pathogenicity *in vivo* by rearranging host cytoskeleton and modulating host cell signaling pathways. The Type 3 Section System is one commonly studied system, but Salmonella species also encode for other secretion systems, which may contribute to pathogenic success (gray secretion systems).Organoid models of the gallbladder support that Salmonella infection directly affects host signaling cascades which increase Salmonella survival and contribute to cellular dysregulation and cancer. Information adapted from (Sepe et al., 2020).

Further mechanistic observations of *Salmonella* association using organoids revealed that infection stimulates cell division and induces phenotypic changes in infected cells by activating the MAPK and AKT (protein kinase B) signaling pathways, leading to changes in cell cycle control (**Figure 3**).

*Salmonella typhi* invasion of epithelial cells resulted in DNA double-strand breaks in the host, which were found to be dependent on the typhoid toxin *cdtB* and that also affected neighboring but non-infected cells (Sepe et al., 2020). *Salmonella typhi* also sustained a long-term infection in induced polarized monolayers at the air-liquid interface, leading to cell cycle arrest independent of the typhoid toxin (Sepe et al., 2020). In contrast, non-infected intoxicated cells continued to proliferate despite sustaining DNA damage. This study underscores the critical role of the typhoid toxin in promoting genomic instability and reinforces the epidemiological link between *Salmonella* infection and GBC (Sepe et al., 2020), paving the way for more exploration of the direct role of bacteria in oncogenesis.

### Lung

Multiple pathogens target vital lung function, such as *Mycobacterium tuberculosis* and *Pseudomonas aeruginosa*, but current air-liquid interface (ALI) monolayer cultures of airway epithelial cells exhibit several limitations, including insufficient mucus production, impaired cilium morphogenesis, disorganized cellular architecture, and limited basal cell renewal. Additionally, some ALI systems are submerged in liquid media, restricting oxygen availability to both bacteria and host cells. In contrast, organoid models overcome these shortcomings in part through apical exposure to the atmosphere, which induces cellular polarization, promotes cilia formation, and supports mucus production. Meanwhile, basal cells at the basolateral side retain their regenerative capacity, further enhancing the physiological relevance of the model. Airway organoids can be used to model early events of tuberculosis infection, including interactions between mycobacteria and epithelial cells, cytokine/antimicrobial responses, and recruitment of macrophages (Sachs et al., 2019).

Human airway organoids have been used to study the early stages of mycobacterial infection. These models revealed that both *M. tuberculosis* and *Mycobacterium abscessus* primarily exist as extracellular bacteria and infect epithelial cells with low infection efficiency, with only 2% of the cells composing the organoid positive for Mycobacteria (Iakobachvili et al., 2022b). These human airway organoid models have also been used to study *Pseudomonas aeruginosa* biofilm formation (Tang et al., 2022). Simultaneous analysis of both bacterial and host transcriptomes from the infected organoid model enabled concurrent observation of the two partner cell types (Tang et al., 2022). This work demonstrated that several protein secretion systems were significantly upregulated in the associated bacteria and, more importantly, quorum sensing (QS) played a crucial role in *P. aeruginosa* pathogenesis and host immune responses, as is observed *in vivo* (Tang et al., 2022).

Other work utilizing live cell imaging of organoids microinjected with *P. aeruginosa* demonstrated that ExoU toxin injected into host cells through the Type-3 Secretion System induced a complete collapse of the organoids, replicating previously observed *in vivo* tissue alteration (Bagayoko et al., 2021). These findings demonstrate that a phospholipase (*exoU*) from *P. aeruginosa* exploits lipid peroxidation to drive pathological effects in human bronchial organoids (Bagayoko et al., 2021). Treatment with Ferrostatin-1 significantly reduced *P. aeruginosa*-induced damage to the organoids (Bagayoko et al., 2021). These advancements in lung organoid technology offer promising avenues for studying pulmonary diseases and developing personalized treatments, as they accurately replicate bacterial-induced cellular changes in the organoid model.

### Urinary tract

Bladder organoids have emerged as valuable models for studying uropathogenic *Escherichia coli* (UPEC) infections, as these 3D structures recapitulate key features of the bladder epithelium (Smith et al., 2008). Hemolysin is understood to be a key player in UPEC infections. To confirm this, a UPEC strain with and without an *hlyA*_1_ mutation was added to monolayers and organoids derived from human bladder 5637 (Smith et al., 2006). The wild-type strain caused significant damage to monolayers, while the organoids retained *in vivo–*like infection response mechanisms (Smith et al., 2006). In contrast, hemolysin deletion in the UPEC strain had minimal impact on both monolayers and organoids, suggesting that hemolysin is a critical factor in causing damage to the urothelium (Smith et al., 2008).

The infection dynamics of another common uropathogen, *Enterococcus faecalis*, have also been evaluated using urothelial organoids. Upon *E. faecalis* infection, organoids exhibited hallmark features of infection, including urothelial cell sloughing and the formation of intracellular bacterial communities (IBCs), a key characteristic of persistent urinary tract infections (UTIs) (Sharma et al., 2021). These intracellular colonies were observed within superficial umbrella cells and resembled pathophysiological events in patient samples (Sharma et al., 2021). Bladder organoids demonstrate that solitary bacteria can invade the bladder wall early, evading antibiotics and neutrophils, independent of intracellular bacterial communities.

### Female reproductive tract

Fallopian tube (FT) organoids have become useful platforms to better understand infection dynamics and host responses to both pathogens of the reproductive tract and to potential probiotic bacteria. *Chlamydia trachomatis* infection of FT organoids resulted in the redirection of host glutamine metabolism to support its replication and for cell wall synthesis (Rajeeve et al., 2020). This metabolic reprogramming was dependent on the c-Myc transcription factor and involved increased glutamine uptake via the SLC1A5 transporter and glutaminolysis (Rajeeve et al., 2020). Follow-on work in mice revealed that interference with glutamine metabolism or knockout of SLC1A5 prevented *chlamydia* from producing progeny, highlighting the central role of glutamine in its pathogenicity (Rajeeve et al., 2020), suggesting that the organoid model accurately led to a key metabolic transition during infection.

Other studies have utilized human FT organoids to investigate chronic *C. trachomatis* infections to gain insight into tissue scarring and infertility. Chronic infection induces changes in host signaling pathways, increases in stemness potential, and accelerates molecular aging within the epithelium (Kessler et al., 2013). These findings suggest that chlamydia infection may contribute to the development of tubal pathologies, including the initiation of high-grade ovarian cancer (Kessler et al., 2013). Researchers also modeled co-infections of Chlamydia and human papillomavirus (HPV) in patient-derived ectocervical organoids, which allowed for the systematic study of individual and co-infection dynamics and provided insights into the interactions between these pathogens and the cervical epithelium (Koster et al., 2022).

Organoid studies have also investigated the probiotic potential of *Lactobacillus crispatus* against the pathogenic activity of *Fannyhessea vaginae* (Yu et al., 2024). FT organoids showed marked differences in inflammatory gene expression during infection with the non-pathogenic *L. crispatus* compared the pathogenic *F. vaginae* (Yu et al., 2024), highlighting the use of organoids in defining microbial influences on reproductive health.

## Limitations

Research on co-infections involving organoids derived from low-biomass tissues with pathogenic and/or commensal bacteria has expanded our understanding of microbial-host interactions. Though an important area of research, the number of studies implementing organoids from low-biomass tissues remains limited, leaving additional questions for the emerging area of low-biomass microbiome association. Existing work primarily focuses on human- and mouse-derived organoids, while *in vitro* models from livestock and companion animals have been largely restricted to investigating intestinal host–microbe interactions (Kawasaki et al., 2022). Moreover, to the best of our knowledge, no studies have examined the interactions between commensal microbiota and organoids derived from low-biomass associations from tissues that are beyond the GIT. Further research is essential to uncover the dynamics and therapeutic implications of such co-infections within these organoid models.

## Conclusion and future directions

The convergence of regenerative medicine and microbiology highlights the critical need to understand the intricate interactions between bacteria and stem cells, a symbiosis that can either enhance or hinder therapeutic outcomes. MSCs, pivotal players in regenerative therapies, are known to interact with microbes present in the body, which can significantly alter their immunomodulatory and regenerative capacities (Kol et al., 2014). Similarly, organoid research has propelled biomedical sciences into a new era, deepening our insights into life, disease, and potential therapies (Wang et al., 2017; Yi et al., 2021). While the initial studies provide hope that organoid models are useful for host/microbiome interaction studies, the reliability and maturity of organoid technology need to be improved for more consistency and expanded to prove their ultimate usefulness.

The existing paradigm linking stem cell properties to regenerative medicine is evolving, necessitating a deeper understanding of the multifaceted role of MSCs as niche cells and tissue organizers (Bianco et al., 2013). Understanding the interplay between microbiome and stem cells is pivotal, as it may hold the key to unlocking more efficacious and predictable regenerative therapies, especially when coupled with advancements in stem cell technologies, tissue engineering, and biomaterials. Understanding these interactions is particularly crucial in the context of tissue engineering, where the presence of bacteria can dramatically influence the success of regenerative approaches (Mhashilkar and Atala, 2012). The complexity of these interactions necessitates thoughtful modeling approaches and careful consideration of *in vivo* recapitulation to understand multifaceted microbial impacts with precision.

This precision offered by organoids also presents an exciting future for host–microbe interaction studies. Well-characterized organoid platforms provide an unprecedented opportunity to evaluate emerging host–microbiome interaction theories. For instance, the oral microbe *Fusobacterium nucleatum* has been found in colorectal tumors from patients, and an increasing number of studies suggest *F. nucleatum* is a driver of colorectal tumor development (Wang and Fang, 2023; Zepeda-Rivera et al., 2024). Though associations between *F. nucleatum* and cancer have been posited, the mechanistic nature of this host–microbe relationship remains understudied in part due to the notable complexity of studying cancer progression *in vivo*. Organoids, which minimize confounding factors while recapitulating many *in vivo* responses, present as a model platform for further studies of oncogenic bacteria. Compounding their usefulness, hybrid organoids provide the additional opportunity to study theorized bacterial-mediated organ axes, such as the gut–brain (Carabotti et al., 2015), mouth–brain (Bowland and Weyrich, 2022), and mouth–urinary connections (Yuan et al., 2021). These organ–organ–microbe connections remain difficult to study in animal models, while other *in vitro* platforms lack the ability to faithfully model multiple systems simultaneously. Organoids thus serve as a middle-ground *in vitro* model, reducing complexity to allow for mechanistic observation while replicating many *in vivo* responses. Further characterization and validation of organoid models will continue to be necessary, especially as more complex organoid experiments are published, but the current utility of organoids in host–microbe interaction work is a promising start.
